# Crystal structure and optical spectroscopic analyses of (*E*)-3-(1*H*-indol-2-yl)-1-(4-nitro­phen­yl)prop-2-en-1-one hemihydrate

**DOI:** 10.1107/S2056989018014329

**Published:** 2018-10-16

**Authors:** Muhamad Fikri Zaini, Ibrahim Abdul Razak, Wan Mohd Khairul, Suhana Arshad

**Affiliations:** a X-ray Crystallography Unit, School of Physics, Universiti Sains Malaysia, 11800 USM, Penang, Malaysia; b School of Fundamental Science, Universiti Malaysia Terengganu, 21030, Kuala Terengganu, Terengganu, Malaysia

**Keywords:** chalcone, crystal structure, DFT, UV–vis, HOMO–LUMO, Hirshfeld surface

## Abstract

The asymmetric unit of the title compound, 2C_17_H_12_N_2_O_3_·H_2_O comprises two mol­ecules of (*E*)-3-(1*H*-indol-2-yl)-1-(4-nitro­phen­yl)prop-2-en-1-one and a water mol­ecule. The main mol­ecule adopts an *s*-*cis* configuration with respect to the C=O and C=C bonds. The dihedral angle between the indole ring system and the nitro-substituted benzene ring is 37.64 (16)°. In the crystal, mol­ecules are linked by O—-H⋯O and N—H⋯O hydrogen bonds, forming chains along [010]. In addition, weak C—H⋯O, C—H⋯π and π–π inter­actions further link the structure into a three-dimensional network. The optimized structure was generated theoretically *via* a density functional theory (DFT) approach at the B3LYP/6–311 G++(d,p) basis level and the HOMO–LUMO behaviour was elucidated to determine the energy gap. The obtained values of 2.70 eV (experimental) and 2.80 eV (DFT) are desirable for optoelectronic applications. The inter­molecular inter­actions were qu­anti­fied and analysed using Hirshfeld surface analysis.

## Abstract 2

The title mol­ecule adopts an *s*-*cis* configuration with respect to the C=O and C=C bonds. The dihedral angle between the indole ring system and the nitro-substituted benzene ring is 37.64 (16)°. In the crystal, mol­ecules are linked by O—-H⋯O and N—H⋯O hydrogen bonds, forming chains along [010]. In addition, weak C—H⋯O, C—H⋯π and π–π inter­actions further link the structure into a three-dimensional network.

## Chemical context   

Chalcone compounds consist of open-chain flavanoids in which two aromatic rings are joined by a three carbon α,β-unsaturated carbonyl system (Thanigaimani *et al.*, 2015 ▸). The design of the chalcone system such as donor–π–acceptor (*D*–π–*A*) plays a significant role in intra­molecular charge–transfer transitions (ICT) in which optical excitation leads to the movement of charge from the donor group to the acceptor group. In addition, the chalcone bridge consists of two different double bonds, C=C and C=O, which contribute to the conjugation of charge transfer, leading to their excellent structural and spectroscopic properties (de Toledo *et al.*, 2018 ▸). Furthermore, the non-linear optical (NLO) properties of chalcone mol­ecules originate mainly from a strong donor–acceptor intra­molecular inter­action and delocalization of the π-electrons (Prabhu *et al.*, 2015 ▸). Many researchers are currently investigating the nitro (NO_2_) group as an acceptor group because the decrease of the resonance effect leads to substantial changes in π-electron delocalization in the ring (Dobrowolski *et al.*, 2009 ▸). In this work, the title chalcone compound was successfully synthesized and its crystal structure is reported herein.
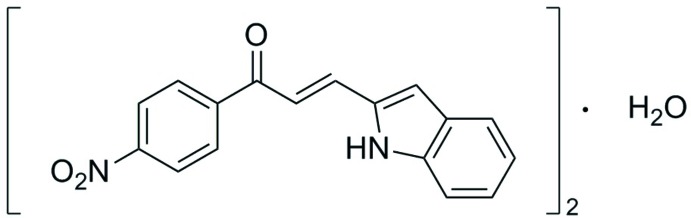


## Structural commentary   

**Figure 1:**
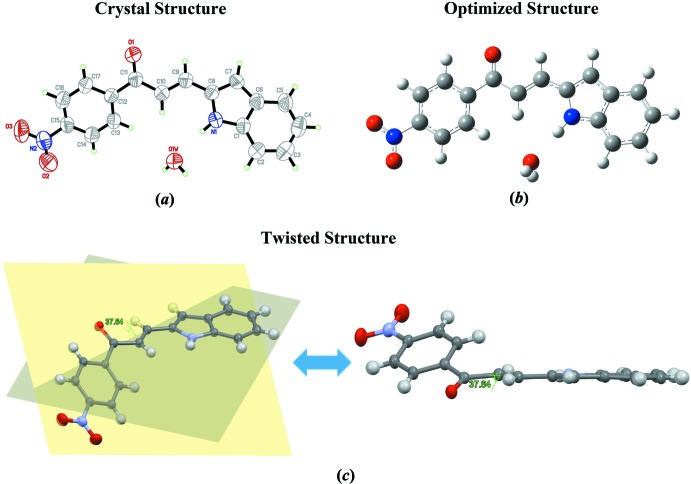
(*a*) The mol­ecular structure of the title compound showing 50% probability ellipsoids, (*b*) the optimized mol­ecular structure and (*c*) a representation of the mol­ecule showing the dihedral angle between the two chosen planes.

The mol­ecular structure of the title compound is shown in Fig. 1 ▸ *a*. The structure was optimized with the *Gaussian09W* software package using the DFT method at the B3LYP/6-311G++(d,p) level, providing information about the geometry of the mol­ecule. The optimized structure is shown in Fig. 1 ▸ *b*. The geometrical parameters are mostly within normal ranges, the slight deviations from the experimental values are due to the fact that the optimization is performed in isolated conditions, whereas the crystal environment and hydrogen-bonding inter­actions affect the results of the X-ray structure (Zainuri *et al.*, 2017 ▸).

In the title compound, the enone group (O1/C9–C11) adopts an *s*-*cis* configuration with respect to the C11=O1 [1.209 (4) Å] and C9=C10 [1.310 (5) Å] bonds. The compound is twisted about the C10—C11 bond with C9—C10—C11—O1 torsion angle of −21.9 (6)°. The corres­ponding torsion angle obtained from the DFT study is 0.08°. In addition, the mol­ecule is twisted about the C11—C12 bond with an O1—C11—C12—C13 torsion angle of 167.7 (4)° (calculated value 179.4°). The differences between the experimental and calculated values show that the inter­molecular hydrogen bond involving the water mol­ecule does not affect the planarity of the compound. A previous study (Zheng *et al.*, 2016 ▸) reported that the inter­molecular hydrogen bond present in the optimized structure stabilizes both the main mol­ecule and the water mol­ecule, which is why we claim that the hydrogen bond does affect the planar conformation in our optimized structure. In the experimental structure, a weak inter­molecular hydrogen bond involving an O atom of the nitro group (Table 1 ▸) may be responsible for the distortion from planarity of the mol­ecule. Furthermore, the twisted nature of this part of the mol­ecule might also be expected because of the steric effects between the carbonyl group and the nitro-substituted benzene ring (Kozlowski *et al.*, 2007 ▸).

The overall conformation of the mol­ecule can be described by the dihedral angle formed by the indole ring system (N1/C1–C8) and the nitro-substituted benzene (C12–C17) ring with a value of 37.64 (16)° (Fig. 1 ▸ *c*). The enone group (O1/C9–C11) with maximum deviation of 0.082 (3) Å at C11 forms dihedral angles of 21.5 (2) and 16.3 (2)° with the indole ring system and the nitro-substituted benzene ring, respectively.

## Supra­molecular features   

**Figure 2:**
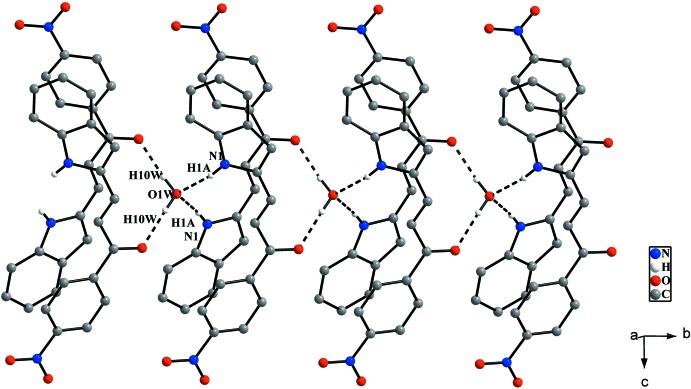
The crystal packing of the title compound along the *b* axis showing the O—H⋯O and N—H⋯O hydrogen bonds as dotted lines.

**Figure 3:**
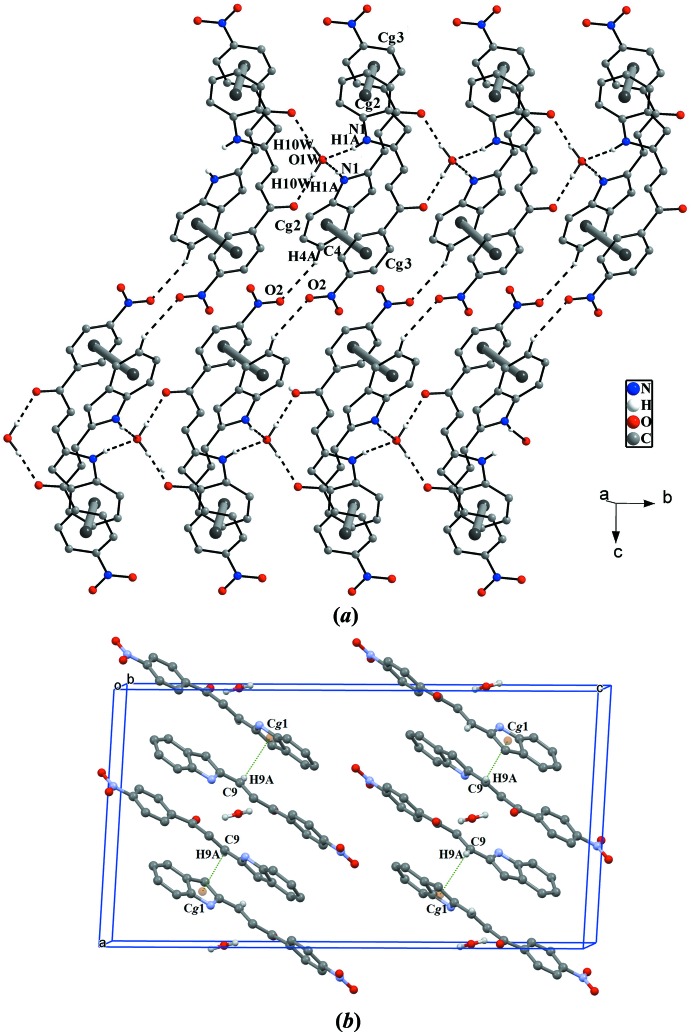
(*a*) A view of the crystal packing showing two of the chains linked by C—H⋯O inter­actions extending along *c*-axis direction. The π–π stacking inter­actions shown by grey lines further stabilize the crystal structure. (*b*) C—H⋯π inter­actions in the title compound. H atoms not involved in hydrogen-bonding inter­actions have been omitted for clarity.

In the crystal, four symmetry-related mol­ecules are connected to each other *via* O—H⋯O and N—H⋯O hydrogen bonds involving the solvent water mol­ecule. The water mol­ecule is connected to the carbonyl group and indole ring system by inter­molecular O1*W*—H1*OW*⋯O1^i^ and N1—H1*A*⋯O1*W* hydrogen bonds (Table 1 ▸), forming chains extending along the *b*-axis direction (Fig. 2 ▸). In addition, weak C4—H4*A*⋯O2^ii^ inter­actions (Table 1 ▸) link these chains into sheets parallel to the *bc* plane (Fig. 3 ▸ *a*). Furthermore, C9—H9*A*⋯*Cg*1 inter­actions (*Cg*1 is the centroid of the N1/C1/C6–C8 ring; Table 1 ▸, Fig. 3 ▸ *b*) are observed along the *a*-axis direction, completing the three-dimensional structure. Two of the anti-parallel mol­ecules are linked by π–π stacking inter­actions (Fig. 3 ▸ *a*) involving the centroids (*Cg*2 and *Cg*3) of the C1–C6 and C12–C17 rings with a centroid–centroid distance *Cg*2⋯*Cg*3 (2-*x*, *y*, 1/2 - *z)* of 3.534 (3) Å*.* These π–π inter­actions further stabilize the crystal structure.

## Hirshfeld surface analysis   

**Figure 4:**
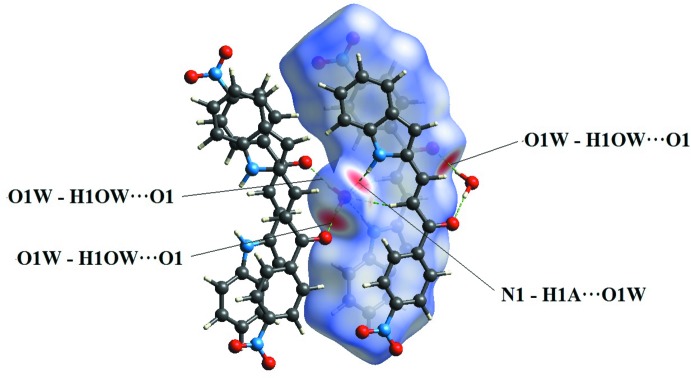
Hirshfeld surface of the title compound mapped over *d*_norm_.

**Figure 5:**
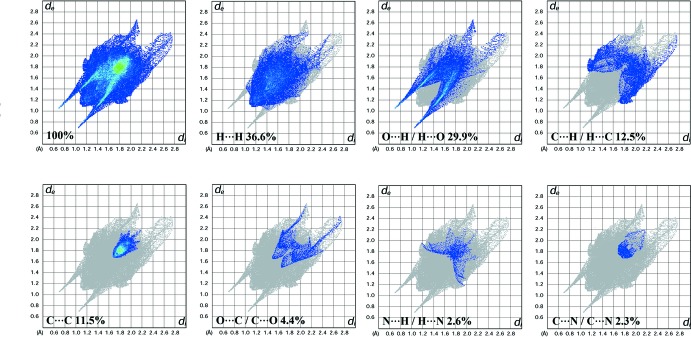
Fingerprint plots of the inter­molecular inter­actions showing the percentage contributions to the total Hirshfeld surface.

Analysis of the Hirshfeld surfaces provides a three-dimensional representation of inter­molecular inter­actions. The Hirshfeld surfaces and related two-dimensional fingerprint (FP) plots were generated with *CrystalExplorer3.1* (Wolff *et al.*, 2012 ▸). In the FP plots, *d*_i_ and *d*_e_ are the distances from the Hirshfeld surface to the nearest atoms outside and inside the surface. The blue colour represents a low frequency of occurrence of a (*d*_i_, *d*_e_) pair and the full fingerprint is outlined in grey (Ternavisk *et al.*, 2014 ▸). The water mol­ecule and H⋯O inter­actions are visualized as bright-red spots on the Hirshfeld surface mapped over *d*_norm_ with neighbouring mol­ecules connected by O1*W*—H1*OW*⋯O1 and N1—H1*A*⋯O1 hydrogen bonds (Fig. 4 ▸). The fingerprint plots indicate the percentage contributions of the various inter­molecular contacts (Fig. 5 ▸). The H⋯H contacts clearly make the most significant contribution (36.6%), whereas O⋯H/H⋯O and C⋯H/H⋯C contacts make contributions of 29.9 and 12.5%, respectively, to the Hirshfeld surface. The presence of O⋯H/H⋯O inter­actions is indicated by two symmetrical narrow spikes with *d*_i_ + *d*_e_ ∼1.7 Å arise specifically due to hydrogen-bonding inter­actions between the water H atom and the carbonyl oxygen. Furthermore, the existence of C⋯H/H⋯C inter­actions is shown by the pair of characteristics wings with the edge at *d*_i_ + *d*_e_ ∼2.9 Å, which is due to the contribution of C—H⋯π inter­action. The 11.5% contribution of the C⋯C inter­actions arises from the π–π inter­action, where the sum of *d*_i_ and *d*_e_ obtained is quite similar at 3.5 Å. Inter­estingly, the N⋯H contacts showed a 2.6% contribution elucidated by a butterfly fingerprint plot resulting from the N1—H1*A*⋯O1 inter­action.

**Figure 6:**
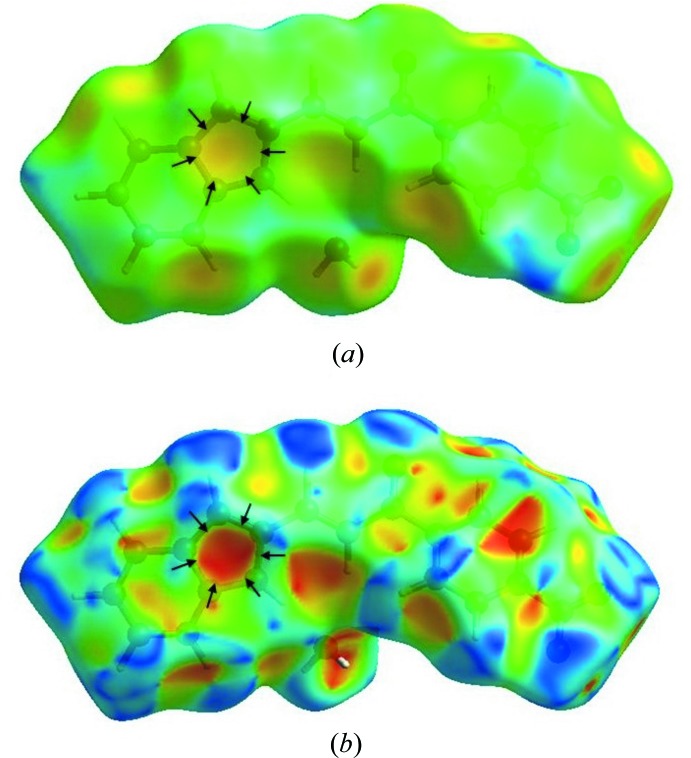
Graphical view of the Hirshfeld surfaces for the title compound (*a*) mapped over *d*_e_ with a pale-orange spot and (*b*) mapped over shape-index with a bright-red spot, both inside the black arrows, signifying the involvement of the C—H⋯π inter­actions.

The presence of the C—H⋯π inter­actions can be seen in the pale-orange spot inside the circle of black arrows on the Hirshfeld surface mapped over *d*_e_ in (Fig. 6 ▸ *a*). With the shape-indexed mapping, the C—H⋯π inter­actions can be observed as a bright-red spot identified with black arrows in Fig. 6 ▸ *b*. The blue spots near the ring represent the reciprocal C—H⋯π inter­actions.

## Frontier mol­ecular orbital and UV–vis studies   

**Figure 7:**
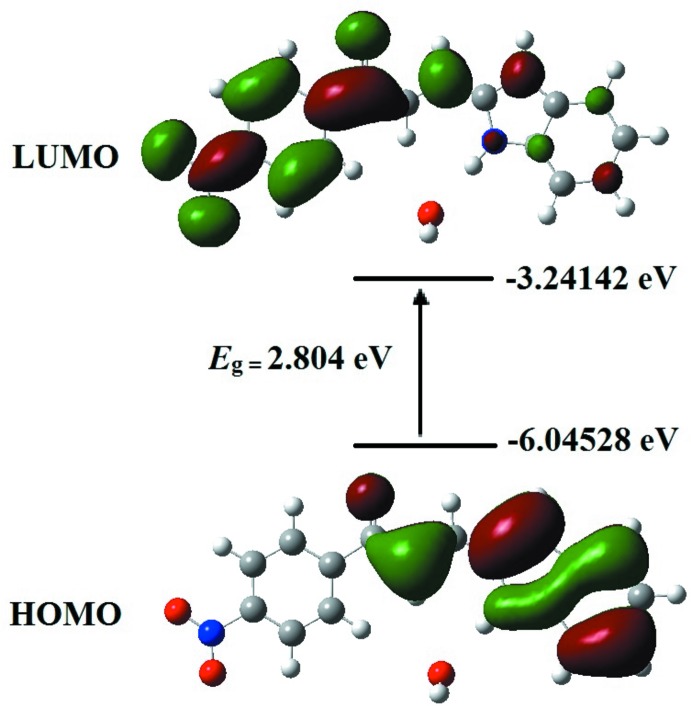
Mol­ecular orbitals showing electronic transition between HOMO–LUMO of the title compound.

Frontier mol­ecular orbital analysis is a vital tool in the development of mol­ecular electronic properties. The energy gap (*E*_g_) between the highest occupied mol­ecular orbital (HOMO) and lowest unoccupied mol­ecular orbital (LUMO) is a crucial factor in elucidating the mol­ecular electrical transport properties. In the present study, the HOMO and LUMO were computed at the DFT/B3LYP/6-311G++(d,p) theoretical level and the respective plots of the frontier mol­ecular orbital are illustrated in Fig. 7 ▸. At a specific separation between donor and acceptor, charge transfer may occur in the ground state if the HOMO of the donor lies energetically above the LUMO of the acceptor (Caruso *et al.*, 2014 ▸). As can be seen from Fig. 7 ▸, the charge at the HOMO state is more localized at the indole group and enone moiety while charge is accumulated entirely at the nitro-substituted phenyl ring and the enone moiety in the LUMO state. The results reveal that the intra­molecular charge transfer (ICT) occurred from the electron-donor groups to the electron-acceptor groups through the enone moiety. The carbon–carbon double bond connecting the donor and acceptor groups is responsible for the charge movement through π-conjugation, triggering electronic delocalization within the mol­ecule (Prabhu *et al.*, 2015 ▸). The energy gap of 2.80 eV obtained from the DFT calculations indicates strong chemical reactivity and weaker kinetic stab­ility, which increase the polarizability and NLO properties (Maidur *et al.*, 2018 ▸).

**Figure 8:**
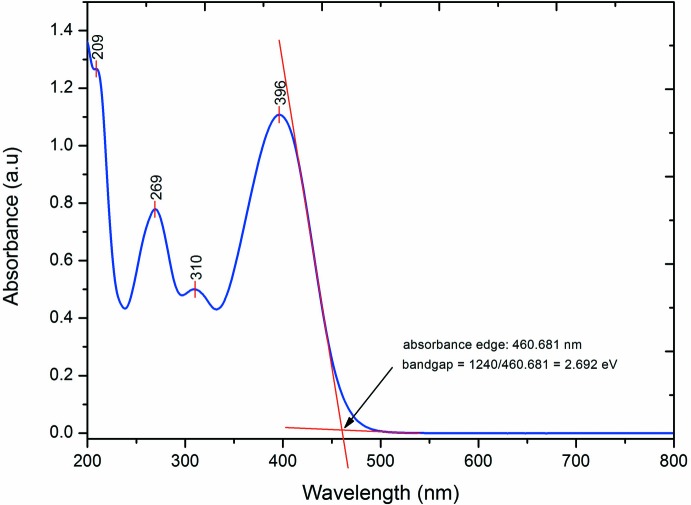
The UV–vis absorption spectrum of the title compound.

The absorption spectrum of the title compound was carried out in aceto­nitrile with a concentration of 10^−4^ *M*. The absorption spectrum comprises of four major bands (Fig. 8 ▸). The strongest band occurs in the region of 396 nm, which was assigned to π–π* transition. This sharp peak is suspected to arise from the indole ring and carbonyl group (C=O). The second strong UV–vis band is observed at 269 nm and is mainly attributed to the electron-withdrawing substituent of the nitro group (Pavia *et al.*, 2001 ▸). The energy gap of the title compound was calculated from the UV–vis absorption edge at 461 nm (Fig. 8 ▸), giving an energy band gap value of 2.70 eV, comparable with the HOMO–LUMO energy gap obtained from the DFT study. This band gap is similar to those in reported studies (D’silva *et al.*, 2011 ▸) and within the energy-gap range for semiconducting materials (Emmanuel *et al.*, 2002 ▸).

## Database survey   

A search of the Cambridge Structural Database (Version 5.39, last update November 2017; Groom *et al.*, 2016 ▸) revealed closely related compounds that differ in the donor substit­uents: 1-(4-nitro­phen­yl)-3-(pyren-1-yl)prop-2-en-1-one (Yu *et al.*, 2017 ▸), 3-(2-fur­yl)-1-(4-nitro­phen­yl)prop-2-en-1-one) (Patil *et al.*, 2006 ▸) and 1-(4-nitro­phen­yl)-3-(2-thien­yl)prop-2-en-1-one (Teh *et al.*, 2006 ▸) with pyrene, furan and thio­phene donor substituent rings. Other related compounds include (2*E*)-3-(2-methyl­phen­yl)-1-(4-nitro­phen­yl)prop-2-en-1-one (Prabhu *et al.*, 2015 ▸) and 3-(4-meth­oxy­phen­yl)-1-(4-nitro­phen­yl)prop-2-en-1-one (Patil *et al.*, 2006 ▸).

## Synthesis and crystallization   

The title compound was synthesized *via* a Claisen–Schmidt condensation reaction. A mixture of 1-(4-nitro­phen­yl)ethan­one (0.5 mmol) and indole-2-carboxaldehyde (0.5 mmol) was dissolved in methanol (20 mL). Sodium hydroxide (NaOH) solution was then added dropwise under vigorous stirring. The reaction mixture was stirred for 5–6 h at room temperature. The final precipitate was filtered, washed with distilled water and recrystallized by slow evaporation from acetone solution to obtain orange plate-shaped crystals.

## Refinement   

Crystal data collection and structure refinement details are summarized in Table 2 ▸. All C-bound H atoms were positioned geometrically (C—H = 0.93 Å) and refined using a riding model with *U*_iso_(H) = 1.2*U*_eq_(C). The water O atom was refined with half-occupancy. The O- and N-bound H atoms were located from difference-Fourier maps and refined freely.

## Supplementary Material

Crystal structure: contains datablock(s) I. DOI: 10.1107/S2056989018014329/lh5883sup1.cif

Structure factors: contains datablock(s) I. DOI: 10.1107/S2056989018014329/lh5883Isup2.hkl

Supporting information file. DOI: 10.1107/S2056989018014329/lh5883Isup3.cml

CCDC reference: 1846181

Additional supporting information:  crystallographic information; 3D view; checkCIF report

## Figures and Tables

**Table 1:** Hydrogen-bond geometry (Å, °) *Cg*1 is the centroid of the N1/C1/C6–C8 ring.

*D*—H⋯*A*	*D*—H	H⋯*A*	*D*⋯*A*	*D*—H⋯*A*
O1*W*—H1*OW*⋯O1^i^	0.88 (4)	1.86 (4)	2.723 (4)	171 (4)
N1—H1*A*⋯O1*W*	0.87 (3)	2.06 (3)	2.923 (4)	170 (3)
C4—H4*A*⋯O2^ii^	0.93	2.60	3.405 (6)	146
C9—H9*A*⋯*Cg*1^iii^	0.93	2.87	3.518 (4)	127

Symmetry codes: (i) 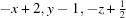; (ii) 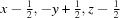; (iii) 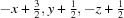.

**Table 2:** Experimental details

Crystal data	
Chemical formula	2C_17_H_12_N_2_O_3_·H_2_O
*M*_r_	602.59
Crystal system, space group	Monoclinic, *C*2/*c*
Temperature (K)	296
*a*, *b*, *c* (Å)	14.835 (7), 6.453 (2), 28.000 (11)
β (°)	93.505 (10)
*V* (Å^3^)	2675.4 (18)
*Z*	4
Radiation type	Mo *K*α
μ (mm^−1^)	0.11
Crystal size (mm)	0.83 × 0.31 × 0.04

Data collection	
Diffractometer	Bruker APEXII CCD
Absorption correction	Multi-scan (*SADABS*; Bruker, 2009 ▸)
*T*_min_, *T*_max_	0.638, 0.955
No. of measured, independent and observed [*I* > 2σ(*I*)] reflections	36005, 2369, 1263
*R*_int_	0.129
(sin θ/λ)_max_ (Å^−1^)	0.595

Refinement	
*R*[*F* ^2^ > 2σ(*F* ^2^)], *wR*(*F* ^2^), *S*	0.061, 0.190, 1.07
No. of reflections	2369
No. of parameters	213
H-atom treatment	H atoms treated by a mixture of independent and constrained refinement
Δρ_max_, Δρ_min_ (e Å^−3^)	0.22, −0.19

Computer programs: *APEX2* and *SAINT* (Bruker, 2009 ▸), *SHELXS97* (Sheldrick 2008 ▸), *SHELXL2013* (Sheldrick, 2015 ▸), *DIAMOND* (Brandenburg, 2009 ▸), *Mercury* (Macrae *et al.*, 2008 ▸) and *PLATON* (Spek, 2009 ▸).

## supplementary crystallographic information

### Crystal data

**Table:**

2C_17_H_12_N_2_O_3_·H_2_O	*F*(000) = 1256
*M**_r_* = 602.59	*D*_x_ = 1.496 Mg m^−^^3^
Monoclinic, *C*2/*c*	Mo *K*α radiation, λ = 0.71073 Å
*a* = 14.835 (7) Å	Cell parameters from 1566 reflections
*b* = 6.453 (2) Å	θ = 2.9–19.5°
*c* = 28.000 (11) Å	µ = 0.11 mm^−^^1^
β = 93.505 (10)°	*T* = 296 K
*V* = 2675.4 (18) Å^3^	Plate, orange
*Z* = 4	0.83 × 0.31 × 0.04 mm

### Data collection

**Table:**

Bruker APEXII CCD diffractometer	1263 reflections with *I* > 2σ(*I*)
φ and ω scans	*R*_int_ = 0.129
Absorption correction: multi-scan (SADABS; Bruker, 2009)	θ_max_ = 25.0°, θ_min_ = 2.8°
*T*_min_ = 0.638, *T*_max_ = 0.955	*h* = −17→17
36005 measured reflections	*k* = −7→7
2369 independent reflections	*l* = −33→33

### Refinement

**Table:**

Refinement on *F*^2^	Hydrogen site location: mixed
Least-squares matrix: full	H atoms treated by a mixture of independent and constrained refinement
*R*[*F*^2^ > 2σ(*F*^2^)] = 0.061	*w* = 1/[σ^2^(*F*_o_^2^) + (0.0548*P*)^2^ + 3.4801*P*] where *P* = (*F*_o_^2^ + 2*F*_c_^2^)/3
*wR*(*F*^2^) = 0.190	(Δ/σ)_max_ < 0.001
*S* = 1.07	Δρ_max_ = 0.22 e Å^−^^3^
2369 reflections	Δρ_min_ = −0.19 e Å^−^^3^
213 parameters	Extinction correction: SHELXL, Fc^*^=kFc[1+0.001xFc^2^λ^3^/sin(2θ)]^-1/4^
0 restraints	Extinction coefficient: 0.0010 (4)

### Special details

**Table:**

Experimental. The following wavelength and cell were deduced by SADABS from the direction cosines etc. They are given here for emergency use only: CELL 0.71150 6.604 8.276 28.665 93.349 89.966 113.537
Geometry. All esds (except the esd in the dihedral angle between two l.s. planes) are estimated using the full covariance matrix. The cell esds are taken into account individually in the estimation of esds in distances, angles and torsion angles; correlations between esds in cell parameters are only used when they are defined by crystal symmetry. An approximate (isotropic) treatment of cell esds is used for estimating esds involving l.s. planes.

### Fractional atomic coordinates and isotropic or equivalent isotropic displacement parameters (Å^2^)

**Table:**

	*x*	*y*	*z*	*U*_iso_*/*U*_eq_	
N1	0.8500 (2)	0.5670 (5)	0.21045 (11)	0.0608 (8)	
H1A	0.892 (2)	0.494 (6)	0.2257 (13)	0.071 (12)*	
N2	1.1251 (3)	0.4606 (7)	0.48886 (12)	0.0826 (11)	
O1	0.98111 (17)	1.0824 (4)	0.33010 (9)	0.0713 (8)	
O2	1.1071 (3)	0.2810 (6)	0.49214 (12)	0.1143 (13)	
O3	1.1754 (2)	0.5484 (6)	0.51669 (11)	0.1137 (12)	
C1	0.8028 (2)	0.5110 (6)	0.16995 (12)	0.0579 (9)	
C2	0.8031 (3)	0.3301 (6)	0.14546 (14)	0.0712 (11)	
H2A	0.8396	0.2200	0.1559	0.085*	
C3	0.7485 (3)	0.3159 (7)	0.10529 (15)	0.0802 (12)	
H3A	0.7470	0.1935	0.0877	0.096*	
C4	0.6954 (3)	0.4782 (8)	0.09007 (15)	0.0802 (12)	
H4A	0.6584	0.4637	0.0622	0.096*	
C5	0.6950 (3)	0.6569 (7)	0.11385 (14)	0.0750 (12)	
H5A	0.6590	0.7666	0.1025	0.090*	
C6	0.7487 (2)	0.6766 (6)	0.15553 (13)	0.0620 (10)	
C7	0.7659 (2)	0.8323 (6)	0.18888 (13)	0.0613 (10)	
H7A	0.7388	0.9623	0.1884	0.074*	
C8	0.8281 (2)	0.7644 (6)	0.22200 (12)	0.0569 (9)	
C9	0.8692 (2)	0.8667 (6)	0.26162 (12)	0.0586 (10)	
H9A	0.8545	1.0055	0.2656	0.070*	
C10	0.9266 (2)	0.7848 (6)	0.29370 (12)	0.0603 (10)	
H10A	0.9386	0.6439	0.2913	0.072*	
C11	0.9717 (2)	0.8965 (6)	0.33203 (12)	0.0574 (9)	
C12	1.0089 (2)	0.7823 (5)	0.37368 (12)	0.0560 (9)	
C13	0.9864 (3)	0.5825 (6)	0.38163 (13)	0.0678 (11)	
H13A	0.9450	0.5162	0.3605	0.081*	
C14	1.0231 (3)	0.4790 (6)	0.41952 (13)	0.0694 (11)	
H14A	1.0072	0.3421	0.4249	0.083*	
C15	1.0831 (2)	0.5757 (6)	0.44943 (12)	0.0634 (10)	
C16	1.1064 (3)	0.7740 (7)	0.44334 (14)	0.0729 (11)	
H16A	1.1475	0.8391	0.4648	0.088*	
C17	1.0683 (3)	0.8770 (6)	0.40510 (13)	0.0668 (11)	
H17A	1.0832	1.0152	0.4004	0.080*	
O1W	1.0000	0.3138 (6)	0.2500	0.0710 (11)	
H1OW	1.012 (3)	0.237 (6)	0.2254 (14)	0.094 (15)*	

### Atomic displacement parameters (Å^2^)

**Table:**

	*U*^11^	*U*^22^	*U*^33^	*U*^12^	*U*^13^	*U*^23^
N1	0.061 (2)	0.063 (2)	0.0561 (19)	0.0041 (17)	−0.0096 (16)	0.0024 (16)
N2	0.090 (3)	0.093 (3)	0.063 (2)	0.004 (2)	−0.010 (2)	0.008 (2)
O1	0.0864 (19)	0.0584 (17)	0.0674 (17)	−0.0039 (14)	−0.0090 (14)	0.0005 (13)
O2	0.153 (3)	0.089 (2)	0.096 (2)	−0.002 (2)	−0.032 (2)	0.025 (2)
O3	0.128 (3)	0.128 (3)	0.079 (2)	−0.006 (2)	−0.043 (2)	0.007 (2)
C1	0.056 (2)	0.064 (2)	0.053 (2)	−0.0047 (18)	−0.0023 (17)	0.0009 (19)
C2	0.079 (3)	0.064 (3)	0.069 (3)	−0.002 (2)	−0.001 (2)	−0.008 (2)
C3	0.081 (3)	0.084 (3)	0.076 (3)	−0.014 (3)	0.006 (2)	−0.018 (2)
C4	0.065 (3)	0.106 (3)	0.068 (3)	−0.011 (3)	−0.008 (2)	−0.015 (3)
C5	0.067 (3)	0.087 (3)	0.069 (3)	0.004 (2)	−0.014 (2)	−0.003 (2)
C6	0.057 (2)	0.067 (2)	0.060 (2)	−0.004 (2)	−0.0039 (18)	0.002 (2)
C7	0.061 (2)	0.056 (2)	0.066 (2)	0.0046 (18)	−0.0078 (19)	0.005 (2)
C8	0.057 (2)	0.056 (2)	0.057 (2)	−0.0013 (18)	−0.0010 (18)	−0.0047 (18)
C9	0.057 (2)	0.060 (2)	0.058 (2)	−0.0015 (17)	−0.0025 (18)	−0.0025 (18)
C10	0.065 (2)	0.056 (2)	0.059 (2)	0.0033 (18)	−0.0023 (19)	−0.0037 (19)
C11	0.055 (2)	0.058 (2)	0.058 (2)	0.0012 (18)	−0.0013 (18)	−0.0020 (19)
C12	0.056 (2)	0.055 (2)	0.056 (2)	0.0008 (18)	−0.0019 (18)	−0.0055 (18)
C13	0.072 (3)	0.065 (2)	0.064 (2)	−0.008 (2)	−0.015 (2)	0.002 (2)
C14	0.077 (3)	0.065 (3)	0.064 (2)	−0.006 (2)	−0.006 (2)	0.002 (2)
C15	0.068 (2)	0.070 (3)	0.051 (2)	0.004 (2)	−0.0066 (19)	0.000 (2)
C16	0.079 (3)	0.076 (3)	0.062 (2)	−0.010 (2)	−0.012 (2)	−0.007 (2)
C17	0.072 (2)	0.064 (2)	0.063 (2)	−0.008 (2)	−0.010 (2)	−0.005 (2)
O1W	0.091 (3)	0.055 (2)	0.065 (3)	0.000	−0.012 (2)	0.000

### Geometric parameters (Å, º)

**Table:**

N1—C1	1.345 (4)	C7—H7A	0.9300
N1—C8	1.359 (4)	C8—C9	1.399 (5)
N1—H1A	0.87 (4)	C9—C10	1.310 (5)
N2—O3	1.189 (4)	C9—H9A	0.9300
N2—O2	1.194 (4)	C10—C11	1.426 (5)
N2—C15	1.440 (5)	C10—H10A	0.9300
O1—C11	1.209 (4)	C11—C12	1.459 (5)
C1—C2	1.354 (5)	C12—C17	1.353 (5)
C1—C6	1.382 (5)	C12—C13	1.353 (5)
C2—C3	1.348 (5)	C13—C14	1.341 (5)
C2—H2A	0.9300	C13—H13A	0.9300
C3—C4	1.363 (6)	C14—C15	1.338 (5)
C3—H3A	0.9300	C14—H14A	0.9300
C4—C5	1.332 (5)	C15—C16	1.339 (5)
C4—H4A	0.9300	C16—C17	1.354 (5)
C5—C6	1.378 (5)	C16—H16A	0.9300
C5—H5A	0.9300	C17—H17A	0.9300
C6—C7	1.385 (5)	O1W—H1OW	0.88 (4)
C7—C8	1.341 (4)		
			
C1—N1—C8	109.4 (3)	N1—C8—C9	122.2 (3)
C1—N1—H1A	126 (2)	C10—C9—C8	126.0 (4)
C8—N1—H1A	124 (2)	C10—C9—H9A	117.0
O3—N2—O2	123.1 (4)	C8—C9—H9A	117.0
O3—N2—C15	118.8 (4)	C9—C10—C11	124.6 (3)
O2—N2—C15	118.1 (4)	C9—C10—H10A	117.7
N1—C1—C2	129.9 (4)	C11—C10—H10A	117.7
N1—C1—C6	107.6 (3)	O1—C11—C10	121.2 (3)
C2—C1—C6	122.6 (3)	O1—C11—C12	119.9 (3)
C3—C2—C1	117.4 (4)	C10—C11—C12	118.9 (3)
C3—C2—H2A	121.3	C17—C12—C13	118.7 (4)
C1—C2—H2A	121.3	C17—C12—C11	119.4 (3)
C2—C3—C4	121.0 (4)	C13—C12—C11	121.9 (3)
C2—C3—H3A	119.5	C14—C13—C12	120.8 (4)
C4—C3—H3A	119.5	C14—C13—H13A	119.6
C5—C4—C3	122.0 (4)	C12—C13—H13A	119.6
C5—C4—H4A	119.0	C15—C14—C13	119.0 (4)
C3—C4—H4A	119.0	C15—C14—H14A	120.5
C4—C5—C6	118.9 (4)	C13—C14—H14A	120.5
C4—C5—H5A	120.6	C14—C15—C16	122.2 (4)
C6—C5—H5A	120.6	C14—C15—N2	118.6 (4)
C5—C6—C1	118.1 (4)	C16—C15—N2	119.2 (4)
C5—C6—C7	135.3 (4)	C15—C16—C17	118.2 (4)
C1—C6—C7	106.5 (3)	C15—C16—H16A	120.9
C8—C7—C6	108.6 (3)	C17—C16—H16A	120.9
C8—C7—H7A	125.7	C12—C17—C16	121.0 (4)
C6—C7—H7A	125.7	C12—C17—H17A	119.5
C7—C8—N1	107.8 (3)	C16—C17—H17A	119.5
C7—C8—C9	130.0 (4)		
			
C8—N1—C1—C2	179.9 (4)	C8—C9—C10—C11	175.8 (3)
C8—N1—C1—C6	−0.6 (4)	C9—C10—C11—O1	−21.9 (6)
N1—C1—C2—C3	−179.9 (4)	C9—C10—C11—C12	159.5 (4)
C6—C1—C2—C3	0.6 (6)	O1—C11—C12—C17	−13.1 (5)
C1—C2—C3—C4	0.2 (6)	C10—C11—C12—C17	165.5 (3)
C2—C3—C4—C5	0.0 (7)	O1—C11—C12—C13	167.7 (4)
C3—C4—C5—C6	−1.2 (6)	C10—C11—C12—C13	−13.7 (5)
C4—C5—C6—C1	1.9 (6)	C17—C12—C13—C14	−0.9 (6)
C4—C5—C6—C7	179.8 (4)	C11—C12—C13—C14	178.3 (4)
N1—C1—C6—C5	178.7 (3)	C12—C13—C14—C15	−0.4 (6)
C2—C1—C6—C5	−1.7 (6)	C13—C14—C15—C16	1.3 (6)
N1—C1—C6—C7	0.2 (4)	C13—C14—C15—N2	−177.3 (4)
C2—C1—C6—C7	179.8 (3)	O3—N2—C15—C14	−176.8 (4)
C5—C6—C7—C8	−177.8 (4)	O2—N2—C15—C14	2.8 (6)
C1—C6—C7—C8	0.3 (4)	O3—N2—C15—C16	4.5 (6)
C6—C7—C8—N1	−0.6 (4)	O2—N2—C15—C16	−176.0 (4)
C6—C7—C8—C9	177.6 (4)	C14—C15—C16—C17	−0.9 (6)
C1—N1—C8—C7	0.7 (4)	N2—C15—C16—C17	177.8 (4)
C1—N1—C8—C9	−177.7 (3)	C13—C12—C17—C16	1.4 (6)
C7—C8—C9—C10	176.4 (4)	C11—C12—C17—C16	−177.9 (4)
N1—C8—C9—C10	−5.5 (6)	C15—C16—C17—C12	−0.5 (6)

### Hydrogen-bond geometry (Å, º)

Cg1 is the centroid of the N1/C1/C6–C8 ring.

**Table:**

*D*—H···*A*	*D*—H	H···*A*	*D*···*A*	*D*—H···*A*
O1*W*—H1*OW*···O1^i^	0.88 (4)	1.86 (4)	2.723 (4)	171 (4)
N1—H1*A*···O1*W*	0.87 (3)	2.06 (3)	2.923 (4)	170 (3)
C4—H4*A*···O2^ii^	0.93	2.60	3.405 (6)	146
C9—H9*A*···*Cg*1^iii^	0.93	2.87	3.518 (4)	127

Symmetry codes: (i) −*x*+2, *y*−1, −*z*+1/2; (ii) *x*−1/2, −*y*+1/2, *z*−1/2; (iii) −*x*+3/2, *y*+1/2, −*z*+1/2.
